# Electron and spin dynamics in a single quantum emitter

**DOI:** 10.1038/s41598-026-44746-4

**Published:** 2026-03-25

**Authors:** F. Rimek, N. Schwarz, H. Mannel, M. Zöllner, A. Ludwig, A. Lorke, M. Geller

**Affiliations:** 1 Faculty of Physics and CENIDE, University of Duisburg-Essen, 47057 Duisburg, Germany; 2 Experimental Physics VI, Faculty of Physics and Astronomy, Ruhr University Bochum, 44801 Bochum, Germany

**Keywords:** Materials science, Nanoscience and technology, Optics and photonics, Physics

## Abstract

The Auger–Meitner effect is a fundamental electron–electron scattering process that impacts the electron and spin dynamics in semiconductor quantum emitters, such as colloidal nanocrystals and quantum dots. Here, we present an experimental study of the magnetic-field dependence of Auger–Meitner recombination and spin-related scattering processes in a single self-assembled InAs quantum dot. Using two-color, time-resolved resonance fluorescence with spectrally separated detection of both exciton and trion transitions, we extract the Auger–Meitner recombination rate, the electron spin-flip relaxation rate, and the spin-flip Raman scattering rate over a broad magnetic-field range from $B = 0$ to $8\,\textrm{T}$. We observe a suppression of the Auger–Meitner recombination rate for magnetic fields above $B = 4\,\textrm{T}$. In contrast, the electron spin-flip relaxation rate increases strongly for fields above $B = 3\,\textrm{T}$ and decreases at lower magnetic fields, while the spin-flip Raman scattering rate remains nearly constant. Our results demonstrate that two-color, time-resolved resonance fluorescence enables access to all relevant microscopic rates for optimizing quantum dots as building blocks for future quantum technologies.

## Introduction

The Auger–Meitner effect^1^ is a fundamental electron–electron scattering process, where the transition energy of an excited electron is transferred to a second electron. While the Auger–Meitner effect was first observed in $\beta$-ray spectroscopy with inner-shell ionization of the atoms^2^, it was later also identified in bulk semiconductors as well as in quantum wells and as an unwanted effect degrading the performance in quantum-optical devices^3,4^. In this sense, it is an important effect in all semiconductor structures, including nanostructures like colloidal nanocrystals^5–8^ and self-assembled quantum dots (QDs)^9–11^. In semiconductor nanostructures that confine carriers in all three spatial dimensions, self-assembled QDs exhibit lateral wave-function extensions on the order of 10–$30\, \textrm{nm}$. This length scale matches the magnetic confinement length $l_B=\sqrt{\hbar /(eB)}\approx 8\,\textrm{nm}$ at experimentally achievable fields of superconducting magnets of $B\approx 10 \,\textrm{T}$, where they can significantly modify the electron and hole wave functions, their orbital energies, and the Coulomb interaction.

Therefore, self-assembled QDs provide an ideal platform for investigating how external magnetic fields modify Auger–Meitner scattering and the intrinsic spin dynamics of a confined charge carrier. Besides Auger–Meitner recombination, the relevant microscopic processes include spin relaxation between the two Zeeman-split electron levels and optically induced spin-flip Raman scattering in magnetic fields. The latter arises predominantly from heavy–light hole mixing in the optically excited trion states^12–16^. In contrast, spin relaxation of the resident electron is governed at low magnetic fields by hyperfine coupling to $10^2$-$10^3$ nuclear spins in CdSe quantum dots^17,18^ and up to the $10^4$–$10^5$nuclear spins in InAs quantum dots^16,19^, while at higher fields ($B \gtrsim 2$T) phonon-assisted spin–orbit interaction becomes the dominant relaxation pathway^19–22^. Experimentally, the spin-relaxation rate in self-assembled InAs QDs has been measured over a wide magnetic-field range: down to a few $10\,\textrm{mT}$ via spin-noise spectroscopy^23^, from 2 to $6\,\textrm{T}$ using time-resolved resonance fluorescence^24^, and from 4 to $10\,\textrm{T}$ in delayed electroluminescence measurements^22^. More recently, Gillard *et al.*^25^ employed a pump–delay–probe resonance fluorescence scheme on InAs quantum dots with different tunnel-barrier thicknesses and extracted spin-relaxation rates up to $B = 8\,\textrm{T}$. They demonstrate electron spin lifetimes exceeding $1\,\textrm{s}$ at $B \approx 0.4\,\textrm{T}$ and show that an effectively isolated dot can reach spin relaxation time with values on the order of tens of seconds, limited by hyperfine-induced relaxation at low fields and phonon-assisted spin–orbit coupling at high fields; three order of magnitude larger than reported in earlier experiments by Lu *et al.*^24^. However, the authors cannot distinguish between the spin-flip Raman scattering from one spin state into the other and the Auger–Meitner recombination into the crystal ground state $\vert {0} \rangle$, as both effects will quench their optically-driven trion transition.

In this work, we aim to provide a comprehensive experimental study of the magnetic field dependence of the Auger–Meitner effect and the spin dynamics in the broad range of the applied magnetic field from $B=0\,\textrm{T}$ up to $8\,\textrm{T}$ in Faraday geometry. We had previously studied the Auger–Meitner scattering rate in time-resolved resonance fluorescence at zero magnetic field^9,26^ and between $B=4$–$10\,\textrm{T}$ in Mannel *et al.*^27^. Here, we now use an optimized experimental setup in resonance fluorescence with a two-laser excitation on the exciton $X^0$ and the negatively charged trion transition $X^-$ in combination with a spectrally resolved detection, simultaneously observing the empty quantum dot $\vert {0} \rangle$ and the dot occupied with one spin-down electron $\vert {\downarrow } \rangle$. This enables us to resolve the individual transition rates that are essential for optimizing self-assembled quantum dots as bright, nearly transform-limited single-photon sources with high indistinguishability^28,29^ and as spin–photon interfaces^30–34^ for quantum networks.

**Fig. 1:**
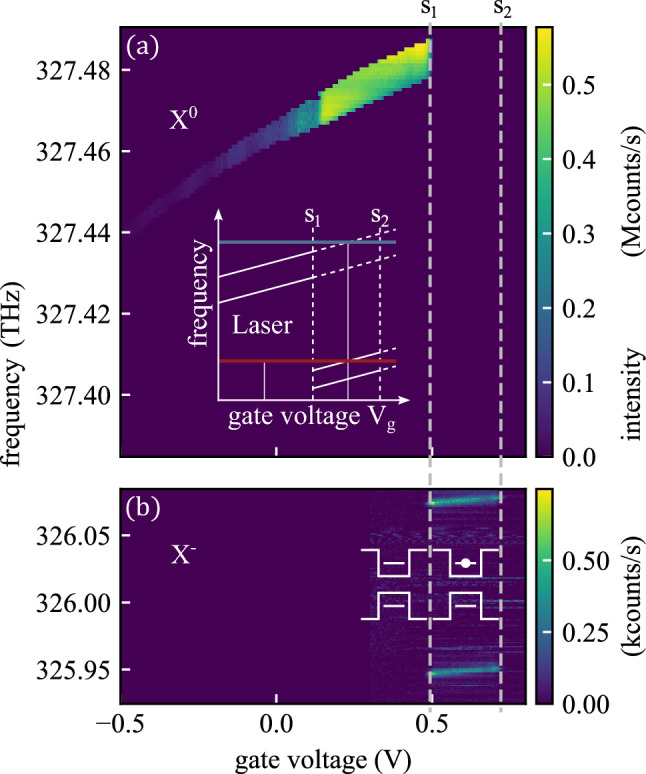
Resonance fluorescence of the QD transitions as a function of gate voltage and laser frequency at an applied magnetic field of $B=4\,\textrm{T}$. (**a**) For gate voltages below $V_\textrm{G}\approx 0.48\,\textrm{V}$, the dot is uncharged and the neutral exciton ($X^{0}$) transition appears at frequencies between $327.440\,\textrm{THz}$ and $327.485\,\textrm{THz}$. The broadening of the exciton line arises from spin dragging (see text). The schematic inset illustrates the principle of the two-laser excitation scheme: one laser (red line) is tuned into resonance with one of the trion transitions, and another laser (blue line) with the exciton transition. The exciton fluorescence is switched on after an Auger–Meitner recombination event empties the dot. (**b**) Between $V_\textrm{G}\approx 0.48\,\textrm{V}$ and $V_\textrm{G}\approx 0.72\,\textrm{V}$, the dot contains a single electron and the two Zeeman-split trion ($X^{-}$) transitions appear at frequencies lower than the exciton resonance. The schematic inset illustrates the corresponding empty dot and the single-electron occupation in the corresponding voltage range.

## Sample structure and resonance fluorescence

The measurements were performed in a confocal microscope setup on a single self-assembled InAs QD in GaAs^35^, embedded in a diode-like heterostructure and cooled in a helium bath cryostat to a base temperature of $T = 4.2\,\textrm{K}$. The sample was grown by molecular beam epitaxy and contains a single layer of QDs with an emission wavelength of about $950\,\textrm{nm}$, enabled by the indium-flush technique applied during the growth procedure^36^. The p–i–n structure consists of a highly n-doped GaAs layer that serves as an electron reservoir and a highly p-doped GaAs layer that acts as the epitaxial gate^27,37^. A 45 nm thick (AlGa)As tunnel barrier between the reservoir and the QD layer ensures an extremely weak tunnel coupling, resulting in tunneling rates on the order of $1/\textrm{ms}$ and a negligible electron exchange by co-tunneling with the reservoir^38,39^. A schematic illustration of our sample structure and details of the experimental setup and the electrical control is shown in supplementary note 1^40^. These tunneling rates are much smaller than those reported in other studies. For example, Gillard *et al.*^25^ observe tunneling rates on the order of $1/\mu \textrm{s}$, which lead to spin randomization through electron exchange with the reservoir and prevents the observation the minimal intrinsic spin relaxation rate. By applying a voltage between the reservoir and the gate, the charge state of the quantum dot can be precisely controlled^41,42^ and the optical transition can be tuned via the quantum-confined Stark effect^43^.

Resonance fluorescence spectroscopy is employed to probe the optical transitions of the quantum dot. A narrow-band continuous-wave laser is tuned into resonance with a transition, and the photons scattered by the emitter are detected on an avalanche photodiode (APD). To resolve the spin and charge dynamics and their transition rates, we use a two-color excitation and detection scheme (see Sec. 4 for details). The strong laser background is suppressed by a cross-polarization technique^44^, where the excitation light is linearly polarized and the detection path is set to the orthogonal polarization (see supplementary note 1).

**Fig. 2:**
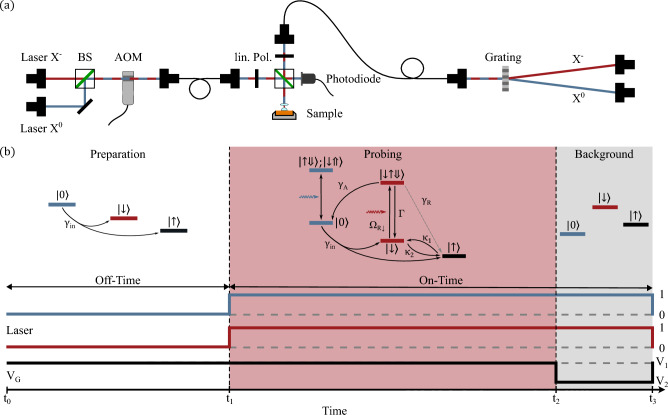
Experimental setup, pulse scheme and quantum states involved in the time-resolved two-color resonance fluorescence measurement. **(a)** Optical setup of the two-laser resonant excitation of the quantum dot in a confocal microscope. A transmission grating is used to separate the emission from the exciton (*X*) and trion ($X^{-}$) transitions. The two resonant lasers are combined on a beamsplitter, and an acousto-optical modulator (AOM) provides nanosecond pulsed excitation. Both laser beams are guided to the dot sample through the microscope inside a cryostat at $4.2\,\textrm{K}$. The emitted photons are spectrally dispersed by a transmission grating and directed onto two avalanche photodiodes (APDs). **(b)** Measurement scheme of the *n*-shot resonance fluorescence experiment. The sequence consists of three phases. In the preparation phase, an electron tunnels into the quantum dot (with rate $\gamma _\textrm{in}$), initializing it in either the spin-up $\vert {\uparrow } \rangle$ or spin-down $\vert {\downarrow } \rangle$ state, such that the trion transition can be driven. In the probing phase, both lasers are switched on and drive the trion or exciton transitions, depending on the charge state of the dot. The trion state can decay via Auger–Meitner recombination $\gamma _\textrm{A}$, spin-flip relaxation $\kappa$, or spin-flip Raman scattering $\gamma _\textrm{R}$. At the end of the probing phase, the gate voltage is set to $V_\textrm{G}<0.48\,\textrm{V}$ to empty the dot and suppress both the exciton and trion transitions, enabling a measurement of the residual laser background and detector dark counts.

## Characterization of the excitonic transitions

In the following, we use the resonance fluorescence technique to characterize the quantum dot with respect to the transition energies of its neutral exciton ($X^{0}$) and negatively charged trion ($X^{-}$) states. Figure 1 shows the resonance fluorescence signal of these transitions in an external magnetic field of $B=4\,\textrm{T}$, plotted as a function of gate voltage and resonant-laser frequency. In a magnetic field, the two optically bright exciton states $\vert {\downarrow \,\Uparrow } \rangle$ and $\vert {\uparrow \,\Downarrow } \rangle$ are split by the spin Zeeman effect. Similarly, the two optically allowed trion transitions connecting the single-electron ground states $\vert {\uparrow } \rangle$ and $\vert {\downarrow } \rangle$ to the corresponding trion states $\vert {\uparrow \downarrow \Uparrow } \rangle$ and $\vert {\downarrow \uparrow \Downarrow } \rangle$ exhibit a Zeeman splitting. This splitting is clearly resolved for the trion transition in Fig. 1(b), where the two allowed branches differ by approximately $100\,\textrm{GHz}$. In contrast, the corresponding splitting of the bright-exciton transition is not visible in Fig. 1(a), because the second Zeeman branch lies outside the scanned frequency range.

In the gate voltage range below $V_\textrm{G} \approx 0.48\,\textrm{V}$, the neutral exciton transition ($X^{0}$) is observed at laser frequencies between $327.440\,\textrm{THz}$ and $327.485\,\textrm{THz}$. In this voltage range, the Fermi level in the electron reservoir is energetically below the electron ground state $s_{1}$ (marked as a vertical dashed line in Fig. 1), and the quantum dot is therefore uncharged (see schematic illustrations in Fig. 1(b)). The exciton transition can then be resonantly driven, here with photon count rate of up to $500\,\mathrm {kCounts/s}$ at an excitation intensity well below the saturation (around 0.05, see supplementary Fig. S4). Spin dragging, i.e., the buildup of nuclear-spin polarization under resonant excitation^45,46^, is visible in Fig. 1(a) as a broadening of the exciton resonance; however, it is not relevant for the measurements discussed in this manuscript. At a gate voltage of $V_\textrm{G} = 0.48\,\textrm{V}$, an electron tunnels into the $s_{1}$ state, and the Zeeman-split trion transitions ($X^{-}$) appear at lower excitation frequencies in Fig. 1(b). A second electron tunnels into the dot at $V_\textrm{G} \approx 0.75\,\textrm{V}$ (vertical dashed line labeled $s_{2}$). For the two-laser excitation scheme discussed in the next section, one laser is tuned into resonance with the energetically lower trion transition at a gate voltage of $V_\textrm{G}=0.57\,\textrm{V}$, where the quantum dot is singly charged (see schematic inset in Fig. 1(a)). The other laser is tuned to the exciton resonance at the same gate voltage. This represents a non-equilibrium situation, because under steady-state conditions the dot is charged with one electron at this voltage and the exciton transition cannot be driven. In this laser excitation configuration, the exciton transition becomes sensitive to the Auger–Meitner recombination, which depopulates the dot via the process $\vert {\downarrow ,\uparrow ,\Downarrow } \rangle \rightarrow \vert {0} \rangle$. The empty dot state |0$\rangle$ can then be excited by the laser that is tuned to the exciton transition until an electron tunnels into the dot from the reservoir.

The exciton resonance can be identified by applying a gate-voltage pulse. An initialization voltage below $V_\textrm{G}=0.48\,\textrm{V}$ is applied, where the dot is empty, before the pulse follows with increasing voltage between the charging thresholds $s_1$ and $s_2$. After pulsing the gate voltage above $V_\textrm{G}=0.48\,\textrm{V}$, the dot remains empty and the exciton transition is visible as long as no electron has tunneled into the dot again with tunneling rate $\approx \textrm{ms}^{-1}$ (see supplementary note 5).

## Time-resolved two-color excitation and detection measurements

To access all transition rates involved in the electron and spin dynamics of an optically driven single quantum dot in a magnetic field, we use two lasers that resonantly address the exciton and trion transitions, respectively, and detect the emitted photons after spectral separation with a diffraction grating. This scheme allows us to distinguish the transitions, gaining more information to improve the accuracy of the later employed rate equation model.

The optical setup is shown in detail in Fig. 2(a). The light from the two lasers (depicted by blue and red lines) are combined into a single optical path via a beamsplitter and coupled into a single-mode fiber. An acousto-optical modulator (AOM) is used to generate pulsed laser excitation. After transmission through the fiber, the beams pass a linear polarizer for laser-background suppression and are focused onto the sample in a confocal microscope geometry. A photodiode is used for stabilization of the laser intensities. The photons scattered from the quantum dot are separated from residual laser light by a second linear polarizer and coupled into an optical fiber. A transmission grating then spectrally separates the emission from the two driven transitions, directing them onto different avalanche photodiodes (APDs).

**Fig. 3:**
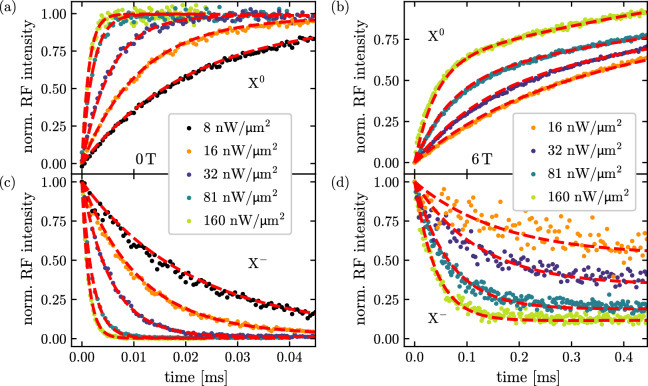
Normalized resonance fluorescence transients of the exciton and trion for increasing laser excitation intensities up to $160\,\mathrm {nW/cm^2}$. The dashed blue ($X^0$) and red ($X^-$) curves represent fits to the experimental data obtained using the rate-equation model (see text). At zero magnetic field, the exciton transient in **(a)** increases with the same rate as the trion transient quenches in **(c)**, since Auger–Meitner recombination is the only mechanism that empties the dot and thus enables resonant excitation of the exciton transition. At a magnetic field of $B = 6\,\textrm{T}$, the rates in the exciton and trion transients in **(b)** and **(d)** differ, as the spin-flip Raman process provides an additional decay channel for the excited trion state (see schematics in Fig. 2). For increasing laser excitation intensity, all transients show a faster dynamics.

Figure 2(b) shows the measurement scheme with the states involved in the upper row during preparation (from $t_0$ to $t_1$), probing (from $t_1$ to $t_2$), and background detection (from $t_2$ to $t_3$). The lower row shows the pulse sequence for both lasers and the applied gate voltage $V_\textrm{G}$. We employ a time-resolved n-shot measurement^27,47^, consisting of up to $10^5$ repetitions to reach a good signal-to-noise ratio in the transients of the exciton *X* and trion $X^-$ transition (see Fig. 3). During the preparation part until time $t_1$, both lasers are switched off, and the gate voltage is set above $V_\textrm{G}= 0.48 \,\textrm{V}$ (see Fig. 1) to ensure that the QD is in the singly charged state. Charging will take place by tunneling from the reservoir with rate $\gamma _\text {in}$, either with an up-spin $\vert {\uparrow } \rangle$ or down-spin $\vert {\downarrow } \rangle$ electron. In this preparation phase (duration of about $t=2\,\textrm{ms}$), the spin states will relax into equilibrium by a phonon-mediated spin-flip process with rate $\kappa$. The occupation probability of spin-up/spin-down then corresponds to a Boltzmann distribution at 4.2 K^13^.

During the probing phase, between $t_{1}$ and $t_{2}$, both lasers are switched on, while the gate voltage is kept constant at $V_\textrm{G}>0.48\,\textrm{V}$. One laser, resonant with the trion transition $\vert {\downarrow } \rangle \rightarrow \vert {\downarrow \uparrow \Downarrow } \rangle$, initiates the spin and charge dynamics in the quantum dot. With Rabi frequency $\Omega _{R\downarrow }$, the trion state $\vert {\downarrow \uparrow \Downarrow } \rangle$ is populated and can decay either into the crystal ground state $\vert {0} \rangle$ through Auger–Meitner recombination (with rate $\gamma _A$), into the spin-down state $\vert {\downarrow } \rangle$ via the optical recombination channel (with rate $\Gamma$), or into the spin-up state $\vert {\uparrow } \rangle$ via spin-flip Raman scattering (with rate $\gamma _\textrm{R}$, see Fig. 2(b)). After Auger–Meitner recombination into the crystal ground state $\vert {0} \rangle$, an electron can tunnel into the dot again. The second laser is resonant with one of the exciton transitions and used to detect the ground state $\vert {0} \rangle$ and thereby provides direct access to the Auger–Meitner recombination rate. At time $t_{2}$, the gate voltage is switched back to $V_\textrm{G}<0.48\,\textrm{V}$, which turns off the trion transition and leaves only laser background and APD dark counts in the detected signal for comparison.

We first consider the case $B = 0\,\textrm{T}$. In the time-resolved *n*-shot measurement, an increasing transient of the exciton ($X^0$) resonance fluorescence intensity is observed in Fig. 3(a), starting from a minimum at $t = 0$ and approaching saturation as dynamic equilibrium is reached after several hundred microseconds. For each transient, the measured background is subtracted, and the resulting signals are normalized such that they directly represent the occupation probability of the exciton or trion state. The trion transition shown in Fig. 3(c) exhibits the opposite behavior: The resonance fluorescence intensity is first at a maximum and then decreases with time.

For increasing trion laser intensity, ranging from $8\,\mathrm {nW/cm^2}$ to $160\,\mathrm {nW/cm^2}$, the trion fluorescence $X^-$ decays faster in Fig. 3(c), while the exciton intensity correspondingly rises faster in Fig. 3(a). At zero magnetic field, the exciton and trion states are not Zeeman split. Consequently, every Auger–Meitner recombination event reduces the electron occupation of the dot and manifests itself as a decrease in the trion resonance fluorescence intensity. At the same time, the probability of finding the dot in its crystal-ground state $\vert {0} \rangle$ increases, which results in a corresponding increase in exciton resonance fluorescence intensity.

Corresponding *n*-shot measurements are performed for magnetic fields up to $10\,\textrm{T}$. For each magnetic field, the time-resolved fluorescence signals are recorded at different laser excitation intensities, spanning from below $\sim \mathrm {nW/cm^2}$ to more than $100\,\mathrm {nW/cm^2}$.

Fig. 3(b) and 3(d) show an example at an applied magnetic field of $B = 6\,\textrm{T}$, where the internal spin and charge dynamics are more complex due to spin-flip relaxation, and spin-flip Raman scattering. As a consequence of the allowed spin-flip Raman process, the trion intensity decreases faster than the exciton fluorescence intensity increases. This is because the trion state $\vert {\downarrow \uparrow \Downarrow } \rangle$ gets an additional decay channel into the spin-up ground state $\vert {\uparrow } \rangle$, as this destroys the resonance condition in the magnetic field. The dashed red lines in Fig. 3 are fits to the data with the rate equation model, as described in the next section. Note here, that the trion and excition transitions are fitted simultaneously with the same fitting parameters.

## Rate equation model

In order to fit the measured transients and derive the different transition rates, the optical transitions associated with the bright exciton and the trion are grouped into effective populations of the exciton state $X^0$ and the trion state $X^{-}$, as these fluorescent optical transition rates (resonant excitation and spontaneous emission) are orders of magnitude faster than the Auger–Meitner, spin-flip and spin-flip Raman scattering rates. This reduction allows the original five level scheme to be mapped onto an effective three level system (see Kleinherbers *et al.*^48^).

The population dynamics of this reduced three level system is governed by incoherent transitions between the exciton state $X^0$, the spin up ground state $\vert {\uparrow } \rangle$ and the trion state $X^{-}$. Since only population probabilities are involved and no optical or spin coherences are present, the system is well described by a classical Markovian rate model (as in Mannel *et al.*^27^). The dynamics can therefore be expressed in compact matrix form by introducing the population vector

1 $$\begin{aligned} \textbf{P}(t)= \begin{pmatrix} P_X(t)\\ P_{\vert {\uparrow } \rangle }(t)\\ P_{X^-}(t) \end{pmatrix}, \end{aligned}$$

which obeys the linear master equation $\dot{\textbf{P}}(t)=\textbf{M}\,\textbf{P}(t)$.

The rate matrix $\textbf{M}$ collects all incoherent processes, including electron tunneling $\gamma _{\textrm{in}}$, Auger–Meitner recombination $\gamma _A$, spin-flips from $\vert {\uparrow } \rangle$ to $\vert {\downarrow } \rangle$ with rate $\kappa _1$ and vice versa with rate $\kappa _2$ and is given by

2 $$\begin{aligned} \textbf{M}= \begin{pmatrix} -\gamma _{\textrm{in}} & 0 & n\,\gamma _A \\ \frac{1}{2}\gamma _{\textrm{in}} & -\kappa _1 & (1-n)\,\kappa _2 + n\,\gamma _R \\ \frac{1}{2}\gamma _{\textrm{in}} & \kappa _1 & -(1-n)\,\kappa _2 + n(\gamma _A+\gamma _R) \end{pmatrix}. \end{aligned}$$

These three rate equations for the effective three-level system, together with the transition rates and the average trion occupation *n* (see supplementary note 4), describe the dynamics of the quantum system shown in Fig. 2(b). For every time *t*, the sum of all state populations $P_{X}$, $P_{\vert {\uparrow } \rangle }$, and $P_{X^-}$ must equal unity. The tunneling rate into the dot, $\gamma _{\textrm{in}}$, is independently determined for each magnetic field in a separate measurement and is provided in the supplementary material. The occupation probabilities of the spin states $\vert {\uparrow } \rangle$ and $\vert {\downarrow } \rangle$ in equilibrium (at the end of the preparation step in Fig. 2(b)) have only a minor influence on the extracted rates and can be estimated assuming a Boltzmann distribution at $T = 4.2\,\textrm{K}$. The energy splitting between the spin-up and spin-down states is approximated from the energy separation of the red and blue trion resonances (see supplementary note 2) as shown in Mannel *et al.*^27^. Using an electron *g*-factor of $g_e = 0.8$ at a transition energy of $1.33\,\textrm{eV}$ and neglecting the smaller hole contribution ($g_h = 0.2$), we obtain thermal occupation probabilities of approximately $65\%$ for $\vert {\uparrow } \rangle$ and $35\%$ for $\vert {\downarrow } \rangle$ at $B = 4\,\textrm{T}$, which increase/decreases to about $93\%$ for $\vert {\uparrow } \rangle$ and $7\%$ $\vert {\downarrow } \rangle$ at $B = 10\,\textrm{T}$.

$\dot{P}_{X^-}(t)$ and $\dot{P}_{X}(t)$ describe the temporal evolution of the normalized resonance fluorescence intensity in Fig. 3 and are numerically integrated, using three fit parameters: the Auger–Meitner rate $\gamma _A$, the spin-flip rate from $\vert {\downarrow } \rangle$ to $\vert {\uparrow } \rangle$, $\kappa _1$, and the spin-flip Raman rate $\gamma _R$. The expressions for $\dot{P}_{X^-}(t)$ and $\dot{P}_{X}(t)$ are used to fit both measured transients simultaneously for each excitation laser intensity and for every applied magnetic field. For $B = 6\,\textrm{T}$ in Fig. 3(b) and (d), the resulting fits are shown as red lines, demonstrating a very good agreement with the experimental data. From these fits, we first get $n \gamma _A$, $n \gamma _R$ and $(1-n) \kappa _2$ in the matrix in Eq. 2 as free fitting parameters, with the occupation probability of the trion state *n*. The actual Auger–Meitner, spin-flip Raman, and spin-relaxation rates are then obtained by division through the trion occupation probability *n*, that we obtained by measuring the saturation curves for every magnetic field (see supplementary note 4).

## Results and discussion

**Fig. 4:**
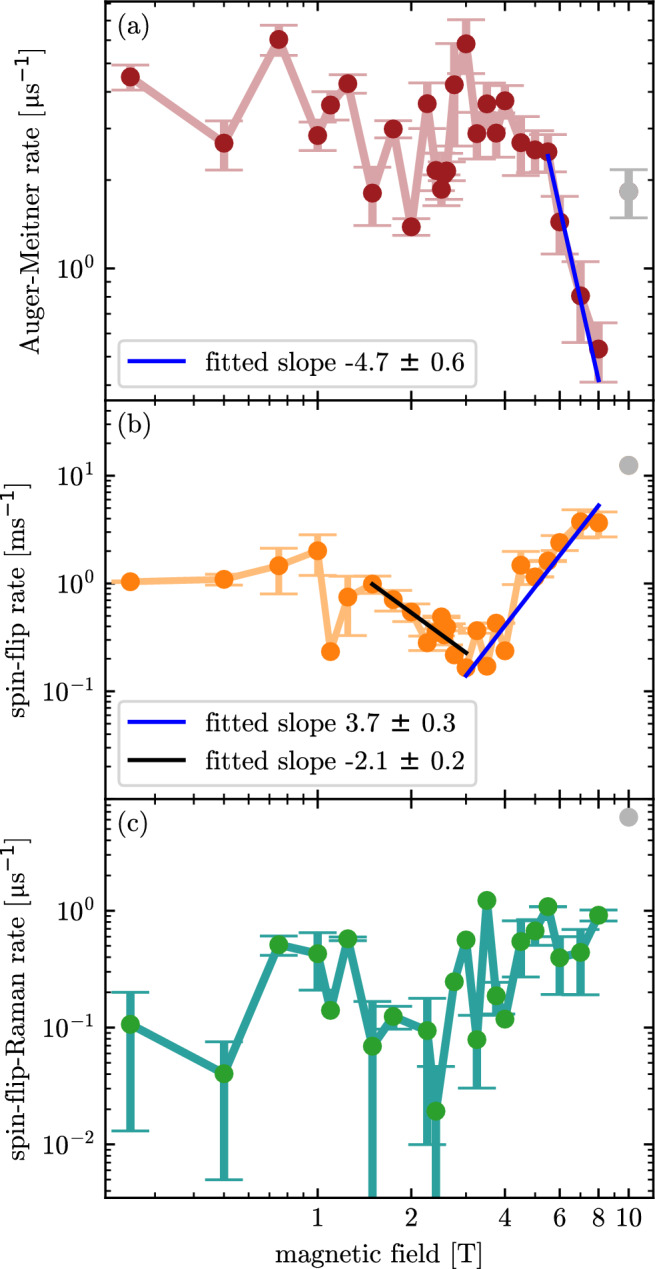
Double-logarithmic representation of the scattering rates extracted from fits to the time-resolved transients in Fig. 3 using the rate-equation model. **(a)** Auger–Meitner rate $\gamma _A$ as a function of the magnetic field, averaged over different trion laser excitation intensities (see text). **(b)** Spin-flip rate $\kappa$ and **(c)** spin-flip Raman rate $\gamma _R$ as a function of the magnetic field. Data points at $B \approx 10\,\textrm{T}$ (grey symbols) are excluded from the quantitative analysis, as no reproducible convergence of the fits is obtained at this magnetic field.

Figure 4 summarizes the averaged scattering rates of the Auger–Meitner process ($\gamma _A$), spin-flip relaxation ($\kappa$), and spin-flip Raman scattering ($\gamma _R$). Since the extracted scattering rates in Fig. 4 remain nearly constant at low trion laser intensities, we average the rates over the intensity range in which no systematic dependence on *n* is observed. For the Auger–Meitner rate, the averaging is restricted to trion occupations up to $n = 0.25$. As shown in the supplementary material, only the spin-flip rate $\kappa$ and the spin-flip Raman rate $\gamma _R$ exhibit an increase at high trion occupations approaching saturation ($n \approx 0.5$). We attribute this behavior to the internal photoeffect^38^, which can lead to photoinduced electron emission from the quantum dot and thereby perturb the rate extraction. Moreover, we observe an atypically strong dependence of the spin-flip and spin-flip Raman rates on the average occupation *n* at $B = 1\,\textrm{T}$. To exclude the influence of such additional unclear perturbations, averaging of the spin-flip and spin-flip Raman rates shown in Fig. 4(b) and (c) is restricted to low trion occupations, i.e. $n < 0.08$. A comparison of averaging up to $n = 0.25$ can be found in supplementary note 3. Data points at $B \approx 10\,\textrm{T}$ are not included in the analysis (gray symbols in Fig. 4), since at this magnetic field the extracted rates show no reproducible convergence. We do not know the origin of missing reproducible convergence. It may indicate that other processes occur with higher magnetic fields that were not present in Mannel et al.^27^ on a different dot, and are not included in the present rate equation model.

The Auger–Meitner rate on a double-logarithmic scale in Fig. 4(a) exhibits weak, non-monotonic variations at low to intermediate fields up to $B = 5.5\,\textrm{T}$ around an average value of $\gamma _A=3\, \mu \text {s}^{-1}$. In a magnetic field, extended electronic states can undergo Landau quantization, which is known to result in an oscillatory density of states and corresponding oscillations in various electrical and optical properties as a function of 1/*B*. The Auger–Meitner rate can generally be described by Fermi’s golden rule, $\gamma _A(B) \propto \sum _f |M_{if}(B)|^2 \rho _f(B)\,\delta \!\bigl (E_i(B)-E_f(B)\bigr )$, where $M_{if}(B)$ denotes the Coulomb matrix element in a magnetic field, and $E_i$ and $E_f$ are the energies of the initial and final states, respectively. Consequently, oscillatory changes in the density of final states $\rho _f(B)$ could translate into changes of the Auger–Meitner recombination rate. Within the experimental uncertainty, however, no clear periodic behavior as a function of 1/*B* can be identified in our present data.

Above a magnetic field of $B = 5.5\,\textrm{T}$, the Auger–Meitner rate decreases monotonically from $\gamma _A = 3\,\mu \mathrm {s^{-1}}$ by a factor of approximately six for increasing magnetic field, reaching $\gamma _A = 0.5\,\mu \mathrm {s^{-1}}$ at $B = 8\,\textrm{T}$. A fit to the data in the double-logarithmic representation (blue line) in the range from $B = 5.5\,\textrm{T}$ to $B = 8\,\textrm{T}$ reveals a power-law dependence $\gamma _A \propto B^{m}$ with an exponent $m = -4.7 \pm 0.6$. However, the value of the exponent is strongly related to the chosen magnetic field range. For instance, using the magnetic field range from $B = 4\,\textrm{T}$ to $B = 8\,\textrm{T}$ results in an exponent of $m = -2.2 \pm 0.4$. The difference in the Auger–Meitner rate for magnetic fields above $B = 4\,\textrm{T}$ in comparison to Mannel et al.^27^ is mainly attributed to a much better resolution in magnetic field and a better accuracy of the measured rate by the two-color excitation technique. Moreover, a different dot on the same sample was used which also could have an influence. Nevertheless, the suppression of the Auger–Meitner rate is evident for both quantum dots in high magnetic fields.

The decrease of the Auger–Meitner rate above $B = 5.5\,\textrm{T}$ cannot be explained within a simple picture of enhanced magnetic confinement increasing the Coulomb matrix element in Fermi’s golden rule. Instead, the final density of states is likely to play an important role in strong magnetic fields due to the above-mentioned Landau-level quantitation. However, a quantitative microscopic understanding of the observed magnetic-field dependence requires theoretical modeling that explicitly incorporates realistic QD wavefunctions, QD potential and the magnetic-field-dependent density of final states, which is beyond the scope of this work. In comparison, the magnetic-field dependence of Auger–Meitner scattering has previously also been reported in photoluminescence measurements of diluted magnetic semiconductor quantum dots^49^. In CdMnSe/ZnSe quantum dots, non-radiative Auger–Meitner-like recombination arises from the interaction between confined electrons and Zeeman-split spin states of Mn ions. The applied magnetic field modifies the Mn spin splitting in these systems. In this magnetic quantum dot system, both the exciton states and the Mn-related levels can be treated within a model of discrete energy levels.

The spin-flip rate $\kappa$ shown in Fig. 4(b) exhibits for magnetic fields above $B = 3\,\textrm{T}$ the well-known power-law dependence on the magnetic field. A fit to the data (blue line) yields an exponent of $m = 3.7 \pm 0.3$, in agreement with the value reported by Gillard *et al.*^25^ and slightly smaller than the theoretical prediction of $m = 5$ for spin–orbit–mediated relaxation^19^. The corresponding spin-flip rates are on the order of $1\,\mathrm {ms^{-1}}$, in good agreement with earlier experimental studies by Kroutvar *et al.*^22^ and Lu *et al.*^24^. These previous experimental studies of the spin-flip rate in InAs/GaAs quantum dots have been conducted at high magnetic fields ($B> 4\,\textrm{T}$). In this magnetic-field range, the spin-flip dynamics is dominated by one-phonon scattering processes that mix the Zeeman-split spin states via spin–orbit interaction^13,19^. Gillard *et al.*^25^ investigated magnetic fields below $B = 3\,\textrm{T}$ in InAs quantum dots and predicted non-monotonic behavior: an initial decrease in the spin-flip rate, followed by an increase due to spin–orbit–mediated relaxation. However, in their experiment, co-tunneling with the reservoir dominates the spin-flip dynamics at low magnetic fields, preventing the observation of this initial decrease in the spin-flip rate as the magnetic field increased. Owing to the very weak tunnel coupling to the electron reservoir, with tunneling rates on the order of $1\,\mathrm {ms^{-1}}$ (see supplementary note 5), we are able here to access the low-field regime $B < 3\,\textrm{T}$ without significant spin randomization due to co-tunneling processes^25^. In this regime, the spin-flip rate decreases from $\kappa \approx 1\,\mathrm {ms^{-1}}$ at $B = 1.5\,\textrm{T}$ to a minimum value of $\kappa \approx 0.2\,\mathrm {ms^{-1}}$ at $B = 3\,\textrm{T}$, corresponding to a power-law slope of $m = -2.1 \pm 0.2$ on a double-logarithmic scale. For magnetic fields below $B < 1\,\textrm{T}$, the spin-flip rate appears to saturate and remains approximately constant at $\kappa \approx 1\,\mathrm {ms^{-1}}$ within the experimental accuracy. Finally, Fig. 4(c) displays the spin-flip Raman rate, which shows no systematic field dependence within the experimental accuracy at a value of about $\gamma _R \approx 0.5\,\mu \mathrm {s^{-1}}$. This value is in reasonable agreement with the spin-pumping rates reported by Lu *et al.*^24^. The weak magnetic-field dependence of $\gamma _R$ reflects the fact that this rate is primarily determined by heavy–light hole mixing in the optically excited states, as discussed by Dreiser *et al.*^13^. Changing the initial occupation of spin-up and spin-down electrons by increasing or decreasing the initial temperature by $\pm 1\,\textrm{K}$ only has a minor effect on the determined scattering rates. The Auger–Meitner rate is affected by less than $1\%$, while the spin-flip and spin-flip Raman rates are affected by less than $3\%$. All scattering rates are systematically increased or decreased with increasing or decreasing temperature, while maintaining the overall dependence on the magnetic field.

## Conclusion

In conclusion, we have presented two-color time-resolved resonance fluorescence measurements with spectrally separated detection of the exciton and blue trion transitions of a self-assembled QD in an applied magnetic field. By resonantly driving both transitions, we investigate the spin and charge dynamics in a weakly tunnel-coupled quantum dot. Our results confirm and extend the findings of Mannel *et al.*^27^ to magnetic fields below $4\,\textrm{T}$ and to a finer magnetic-field resolution. We observe a non-monotonic magnetic-field dependence of the spin-flip relaxation rate, with a decrease at low magnetic fields followed by the well-known increase at higher fields, whereas the spin-flip Raman rate $\gamma _R$ has no observed field dependence over the investigated field range. In contrast, the Auger–Meitner recombination rate $\gamma _A$ remains approximately constant within the experimental accuracy before decreasing between $B= 5.5\,\textrm{T}$ and $B= 8\,\textrm{T}$ by a factor of six, reaching a minimum value of $\gamma _A = 0.5\,\mu \mathrm {s^{-1}}$. Despite this reduction, Auger–Meitner recombination remains at least two orders of magnitude faster than spin relaxation and can substantially limit the electron spin lifetime in an optically excited quantum dot. While the experimental data clearly demonstrate a magnetic-field-dependent change of the Auger–Meitner recombination, a quantitative microscopic model describing the underlying Coulomb scattering processes is presently not available, which represents a limitation of the current work. Our results highlight the importance of suppressing Auger–Meitner processes through advanced quantum-dot heterostructure design for future spin-based quantum devices.

## Supplementary material

The supplementary material consists of additional measurements that are mentioned in the text.

## Supplementary Information

Supplementary Information.

## Data Availability

The datasets generated during and analysed during the current study are available in the DuEData repository, under 10.71955/DUEDATA-2026-V8TMUF.
