# Supplementary material for: Electron and spin dynamics in a single quantum emitter
Source: Sci Rep. 2026 Mar 25;16:10498. doi: 10.1038/s41598-026-44746-4 (PMC13031950; doi:10.1038/s41598-026-44746-4)
Supplement: Supplementary file 1 — Supplementary Information. [file 41598_2026_44746_MOESM1_ESM.pdf]

Supplementary Material: Magnetic-field dependence of the Auger-Meitner recombination and spin dynamics in a single quantum emitter

F. Rimek,[*,†] N. Schwarz,[†] H. Mannel,[†] M. Zöllner,[†] A. Ludwig,[‡] A. Lorke,[†] and M. Geller[†]

†*Faculty of Physics and CENIDE, University of Duisburg-Essen, 47057 Duisburg, Germany*

‡*Experimental Physics VI, Faculty of Physics and Astronomy, Ruhr University Bochum, 44801 Bochum, Germany*

E-mail: fabio.rimek@uni-due.de

Supplementary note 1: sample structure

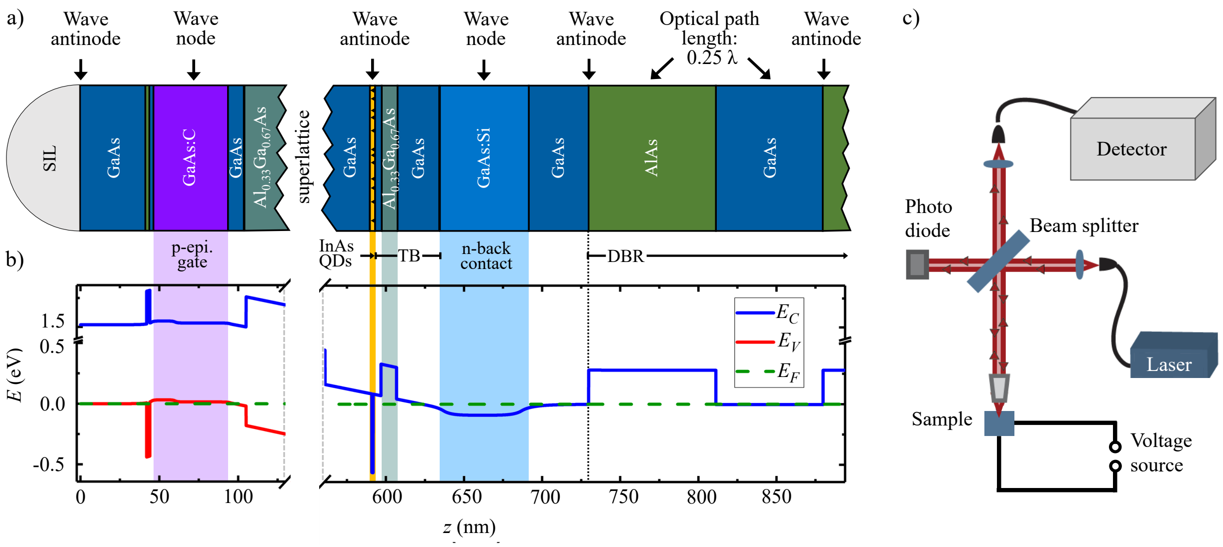

Fig. S1: **Sample structure and calculated band structure.** (a) Starting from the right-hand side, the diode structure consist of a distributed Bragg reflector (DBR), a back contact (GaAs:Si), a tunnel barrier (45 nm GaAs/$Al_{0.33}Ga_{0.67}As$/GaAs), the quantum dot layer (QDs), an AlGaAs/GaAs superlattice, an epitaxial gate (GaAs:C) and a final GaAs layer. The solid immersion lens (SIL) on top of the sample increases the collection efficiency. (b) The conduction band edge E_C is represented with a blue line, while the valence band edge E_V is shown in red. The Fermi energy E_F is indicated by a dashed green line. (c) The confocal microscope consist of a 10/90 beam splitter reflects the laser light down to the sample, which is cooled to 4.2 K in a bath cryostat. There, the light is focused to a 1 μm diameter spot and addresses a single QD. The QD- and reflected laser photons are then coupled into a fiber and guided to the detection path. Adapted from Lochner et al.[1]

Figure S1 schematically illustrates the sample structure, shown from bottom (right side) to top (left side). The conduction-band edge E_C, valence-band edge E_V, and the Fermi energy E_F are depicted in Fig. S1 (b). The corresponding band profile was calculated using a one-dimensional Poisson solver. Figure S1 is adapted from Ref. Lochner et al.[1]

The sample consists of a distributed Bragg reflector (DBR), an electron reservoir, a tunneling barrier, self-assembled quantum dots (QDs), a superlattice, and a top gate. The DBR comprises 16 alternating GaAs and AlAs layers and enhances the optical coupling between the QDs and the excitation laser, thereby increasing the photon collection efficiency. The back contact is formed by a 50 nm thick, heavily Si-doped GaAs layer (light blue,

$z \approx 650\,\text{nm}$), which acts as a three-dimensional electron reservoir and electrical back contact.

The QDs are weakly tunnel-coupled to this reservoir via a barrier consisting of 30 nm GaAs, 10 nm AlGaAs, and 5 nm GaAs ($z \approx 600\,\text{nm}$). Above the QD layer, an AlAs/GaAs short-period superlattice separates the dots from a 45 nm thick C-doped GaAs layer that serves as the top gate. The superlattice primarily acts as a current-blocking barrier; additionally, it suppresses impurity segregation during growth and improves interface smoothness. After growth and device processing, a solid immersion lens (SIL) is placed on top of the structure to enhance optical collection.

Supplementary note 2. Zeeman splitting

In the absence of an external magnetic field, the trion state X^- is degenerate. An applied magnetic field lifts this degeneracy, resulting in a splitting of the trion into the red (X_R^-) and blue (X_B^-) trion states. These labels refer to the transition energies, with the red (blue) trion corresponding to the lower- (higher-) energy transition. The transition energies depend on the magnetic-field strength and are governed by the Zeeman effect. This magnetic-field dependence is shown in Fig. S2, where the transition energies are represented by the excitation laser frequency. The exciton resonance exhibits a similar Zeeman-induced energy shift.

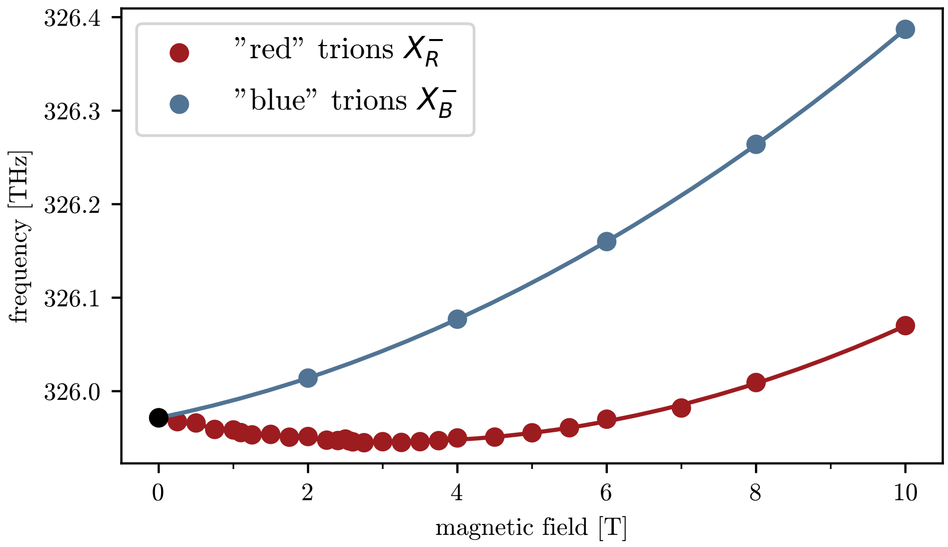

Fig. S2: **Zeeman splitting of trion transitions in a magnetic field.** At $B = 0\,\mathrm{T}$, the trion states are degenerated. With increasing magnetic field, this degeneracy is lifted and the trion splits into a "red" (lower energy) and a "blue" trion (higher energy) transition.

Supplementary note 3. Transition rates and their dependence on the trion excitation intensity

Figure S3(a), (b), and (c) show the three different rates, namely the Auger–Meitner recombination rate, the spin-flip relaxation rate, and the spin-flip Raman scattering rate, as functions of the applied magnetic field. In contrast to the main text, the data are presented here on a linear scale. Panels (b) and (c) display the rates for two different ranges of the excitation intensity of laser 1 driving the trion transition. An increasing excitation intensity of laser 1 corresponds to an increasing average occupation of the trion transition, denoted by n, which governs both the spin-flip Raman scattering rate and the Auger–Meitner recombination rate.

Figure S3(d), (e), and (f) show the different scattering rates as functions of the average trion occupation probability n for increasing magnetic fields from $B = 0.25\,\mathrm{T}$ (blue dots and lines) up to $B = 5.5\,\mathrm{T}$ (violet dots and lines). These measurements were performed to verify that the extracted scattering rates are independent of the occupation probability n. This is clearly the case for occupations up to $n \approx 0.25$.

Accordingly, the Auger–Meitner recombination rates shown in Fig. S3(a) are obtained by averaging over the range $0 < n < 0.25$, indicated by the red shaded area in Fig. S3(d), in order to improve the statistical accuracy. Figures S3(e) and (f) show the spin-flip and spin-flip Raman scattering rates as functions of the trion occupation probability n for the same set of magnetic fields. Both rates remain nearly independent of n over a wide range; however, an increase is observed for occupations above $n \geq 0.3$. This behavior may originate from electron scattering induced by the internal photoeffect, as previously reported by Lochner *et al.*[2]

To avoid this regime, we restrict the averaging to very low occupation probabilities, $n < 0.08$, shown as orange dots and lines in Fig. S3(b) for the spin-flip rate and as green dots and lines in Fig. S3(c) for the spin-flip Raman rate. For comparison, averaging over the range $0.08 < n < 0.25$ is shown as gray dots and lines in Fig. S3(b) and (c). Within the experimental uncertainty, both averaging windows yield identical spin-flip and spin-flip

Raman rates. The only exception is a single outlier in the spin-flip rate at approximately $B = 1\,\mathrm{T}$, for which we currently have no conclusive explanation.

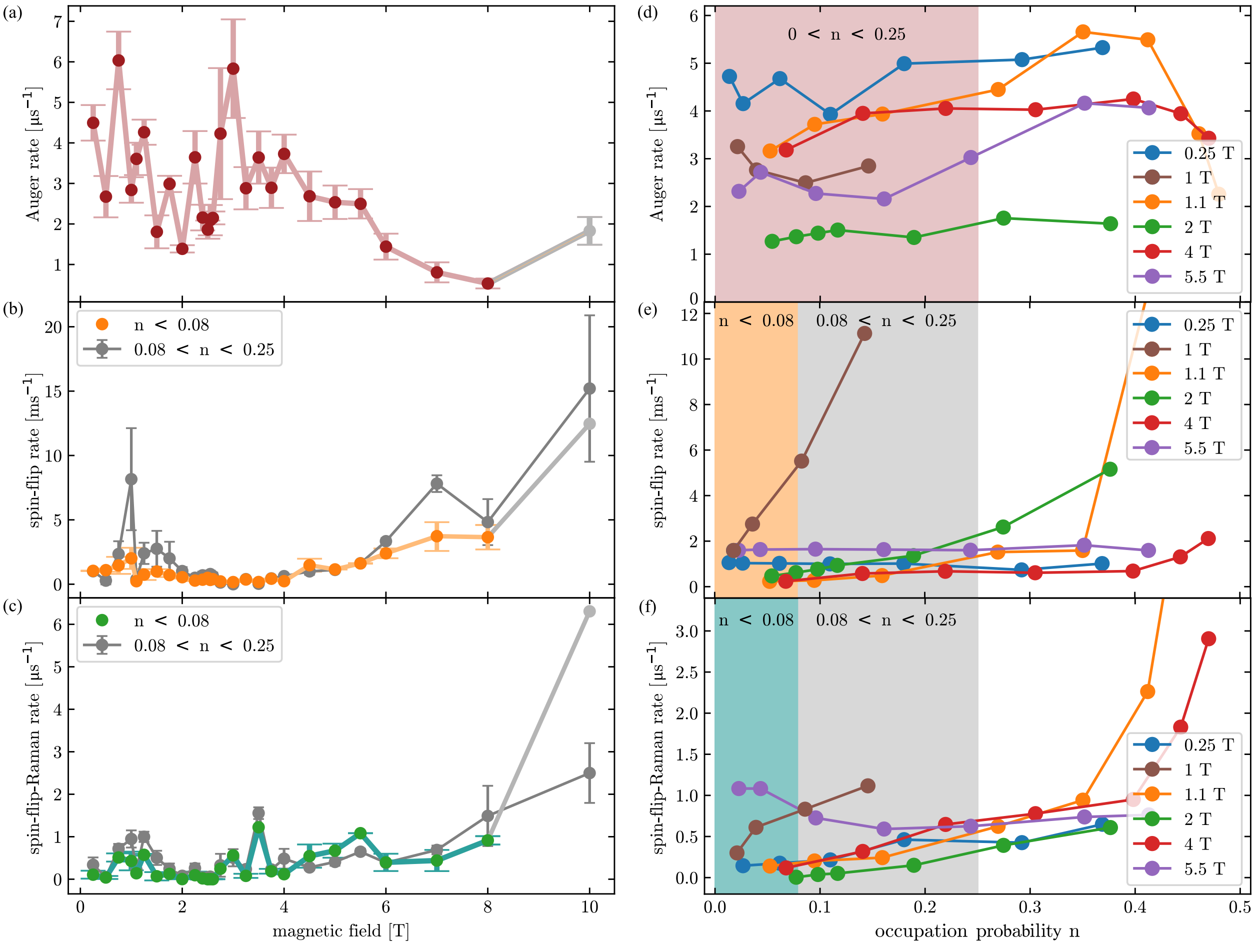

Fig. S3: **Magnetic field and occupation dependence n of the obtained scattering rates.** (a)–(c) Auger–Meitner recombination rate, electron spin-flip relaxation rate, and spin-flip Raman scattering rate as functions of the applied magnetic field, shown on a linear scale. Panels (b) and (c) display the rates for two different ranges of the excitation intensity of laser 1, driving the trion transition, corresponding to an increasing of average trion occupation probability n. (d)–(f) Scattering rates as functions of n for magnetic fields ranging from $B = 0.25\,\mathrm{T}$ (blue) to $B = 5.5\,\mathrm{T}$ (violet), demonstrating that all rates are independent of n up to $n \approx 0.25$. The Auger–Meitner recombination rates in (a) are obtained by averaging over $0 < n < 0.25$, indicated by the red shaded area in (d). For the spin-flip and spin-flip Raman rates, averaging over $n < 0.08$ (colored symbols) and $0.08 < n < 0.25$ (gray symbols) yields identical results within experimental uncertainty.

Supplementary note 4. Saturation curve of the trion state

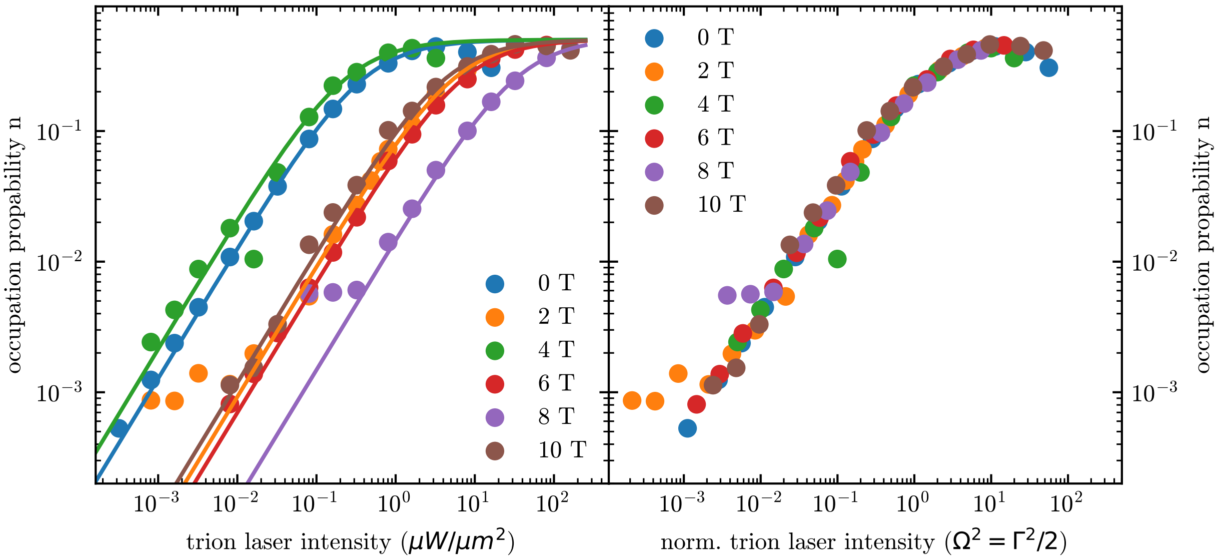

Fig. S4: **Saturation behavior of the trion transition for different magnetic fields.** (a) Average occupation probability n of the excited trion state as a function of the trion laser intensity for magnetic fields between 0 and 10 T. Solid lines are fits to the daty using equation S.1 (see text). (b) Same data plotted as a function of the normalized trion laser intensity, expressed in terms of the Rabi frequency $\Omega^2 = \Gamma^2/2$, demonstrating a collapse of the saturation curves for different magnetic fields on the same data points.

The optical transitions between the excited trion state and the trion ground state occur on timescales that are orders of magnitude faster than the three scattering processes investigated here. For the purpose of our rate-equation model, both trion states can therefore be treated as a single effective state. The occupation probability n of the excited trion state is nevertheless used to parametrize the optical excitation strength and to distinguish between low- and high-excitation regimes. The occupation n is obtained by measurements of the resonance fluorescence intensity of the trion to the excitation intensity of the trion laser for different magnetic fields, as shown in Fig. S4.

This dependence can be described by the so-called saturation equation from the optical Bloch equations with radiative lifetime T_1, dephasing time T_2, Rabi-Frequency Ω and the detuning of the laser energy with respect to the resonance energy $\Delta\omega$ (where $\Delta\omega = 0$ in our

case):

$$n = \frac{1}{2} \frac{\Omega^2 T_1/T_2}{\Delta\omega^2 + 1/T_2^2 + \Omega^2 T_1/T_2} \tag{S.1}$$

Saturation is defined as an average occupation n of $n = 0.25$, corresponding to half of the maximum population of $n = 0.5$ in a regime of infinity high laser excitation of a two-level system. The trion laser intensity required to reach saturation depends on the optical alignment and therefore differs for each magnetic field, as shown in the left panel of Fig. S4. To account for this variation, the excitation strength is expressed in terms of the normalized trion laser intensity, given by the Rabi frequency Ω. The right panel shows, hence, the same data plotted as a function of the normalized trion laser intensity, $\Omega^2 = \Gamma^2/2$, for which the saturation curves obtained at different magnetic fields collapse onto a single curve. The laser intensity that drives the exciton transition was constant during each measurement around $0.08\,\mu W/\mu m^2$.

Supplementary note 5. Magnetic-field dependence of the electron tunneling rate

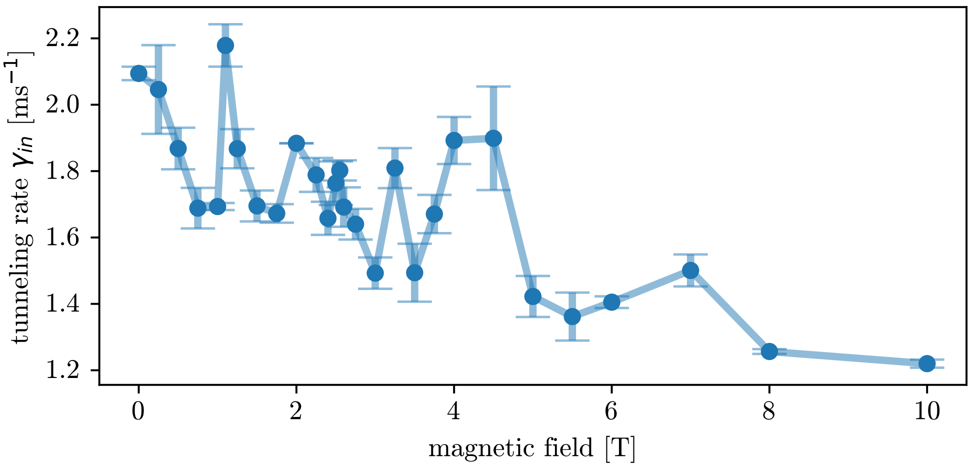

Fig. S5: **Tunneling rate γ_{in} of an electron into the QD as a function of the magnetic field.** This value is determined for each magnetic field in a separate measurement. In this measurement, the exciton transition can be driven by the laser at gate voltages above $V_G = 0.57\,\mathrm{V}$, corresponding to the trion charging regime. By first applying a gate voltage below $V_G = 0.57\,\mathrm{V}$, the quantum dot is emptied. Subsequently pulsing the gate voltage to values above $V_G = 0.57\,\mathrm{V}$ enables the observation of the tunneling of a single electron, which manifests as a quenching of the resonance fluorescence signal.

The rate γ_{in} for electrons to tunnel from the charge reservoir into the quantum dot (QD) was determined by a separate time-resolved resonant fluorescence (RF) n-shot measurements for every magnetic field.[3] The pulse scheme for the n-shot measurements is provided in Mannel et al.[4] Throughout the measurement, the excitation laser remains continuously on. The laser frequency is tuned to the exciton transition at a gate voltage above $V_G = 0.57\,\mathrm{V}$, corresponding to the trion charging regime. At this gate voltage, the exciton transition is forbidden in equilibrium because the QD is occupied by a single electron. To initialize the measurement, the QD is first emptied by setting the gate voltage to $V_G = 0\,\mathrm{V}$, where the Fermi energy in the reservoir is below the s-shell of the dot. At t = 0, the gate voltage is switched back to 0.57 V, where the exciton transition is in resonance with the laser frequency until an electron has tunneled into the QD and the resonance fluorescence intensity quenches.

References

(1) Lochner, P.; Kurzmann, A.; Schott, R.; Wieck, A. D.; Ludwig, A.; Lorke, A.; Geller, M. Contrast of 83% in reflection measurements on a single quantum dot. *Sci. Rep.* **2019**, *9*, 8817.

(2) Lochner, P.; Kerski, J.; Kurzmann, A.; Wieck, A. D.; Ludwig, A.; Geller, M.; Lorke, A. Internal photoeffect from a single quantum emitter. *Phys. Rev. B* **2021**, *103*, 075426.

(3) Amasha, S.; MacLean, K.; Radu, I. P.; Zumbühl, D. M.; Kastner, M. A.; Hanson, M. P.; Gossard, A. C. Spin-dependent tunneling of single electrons into an empty quantum dot. *Physical Review B* **2008**, *78*, 041306.

(4) Mannel, H.; Kerski, J.; Lochner, P.; Zöllner, M.; Wieck, A. D.; Ludwig, A.; Lorke, A.; Geller, M. Auger and spin dynamics in a self-assembled quantum dot. *J. Appl. Phys.* **2023**, *134*.
